# Supplementary material for: Using a Quantitative High-Throughput Screening Platform to Identify Molecular Targets and Compounds as Repurposing Candidates for Endometriosis
Source: Biomolecules. 2023 Jun 8;13(6):965. doi: 10.3390/biom13060965 (PMC10296532; doi:10.3390/biom13060965)
Supplement: Supplementary file 1 [file biomolecules-13-00965-s001.zip › biomolecules-2337989-supplementary.pdf]

biomolecules

MDPI

Supplementary Materials

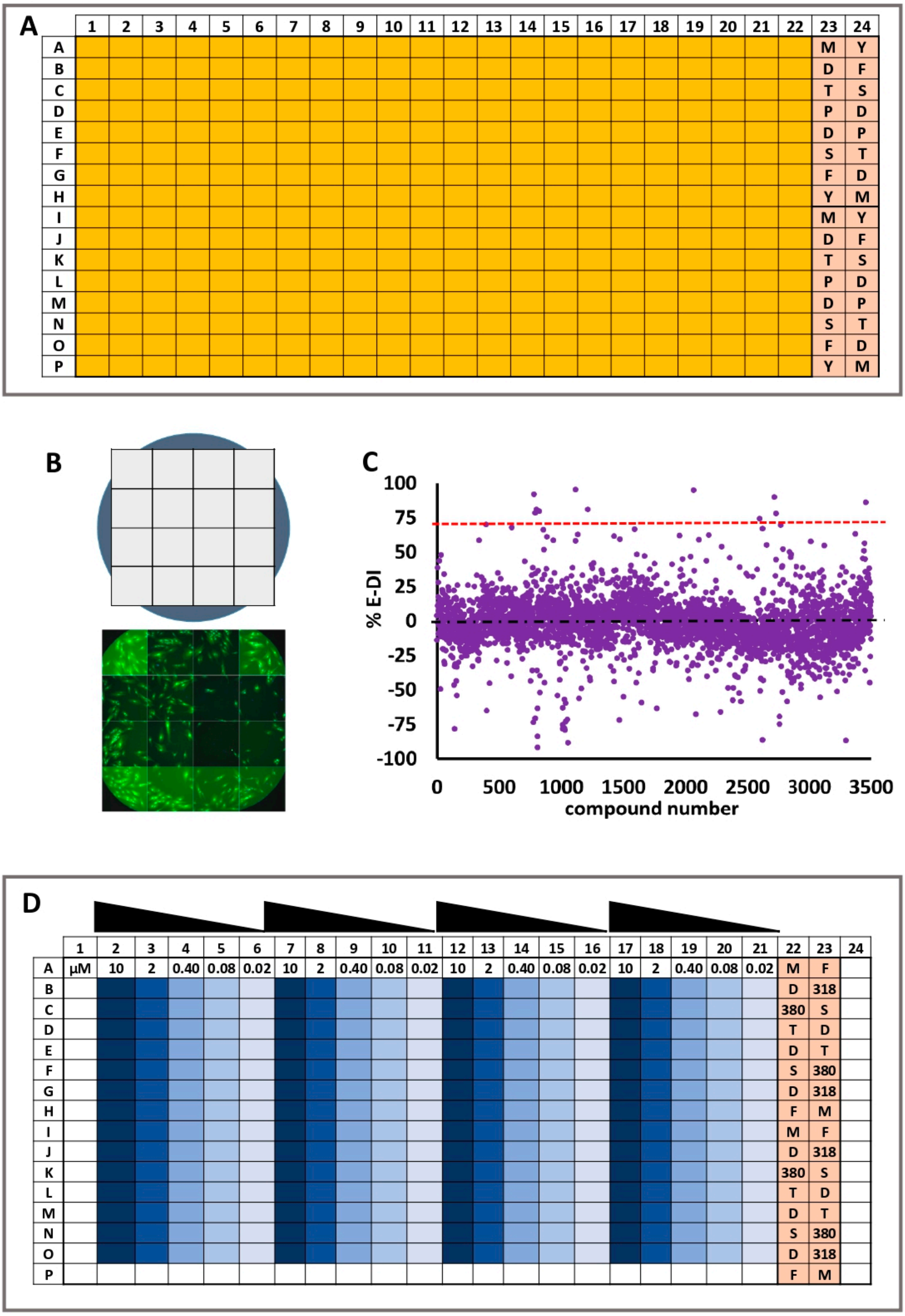

Supplementary Figure S1: Quantitative High Throughput Screening (qHTS) experimental set up. The initial qHTS consisted of a 384 well set up that included 352 wells containing a single compound [10 µM] and columns 23 and 24

contained randomly deposited QC compounds [10 μM] (A). High Content Images (HCI) were taken using a 16-square array at 10X magnification to capture entire well (B). Cells stained green with CMFDA. The %E-DI compound response representation to show the large variability in cellular response to the compounds screened (C). Compounds above zero inhibited cell growth and compounds above 70% (red dotted line) were selected for further investigation. In the final qHTS, the hit confirmation consisted of a 384 well set up where a single compound was serially diluted 1:5 over 5 wells [10-0.02 μM]. Column 22 and 23 contained a single dose of QC compounds [10 μM] (D). QC compounds listed were media (M), DMSO (D), tamoxifen (T), fulvestrant (F), salinomycin (S), panobinostat (P), Y26732 (Y), SNO1006318 (318), SNO1004380 (380).

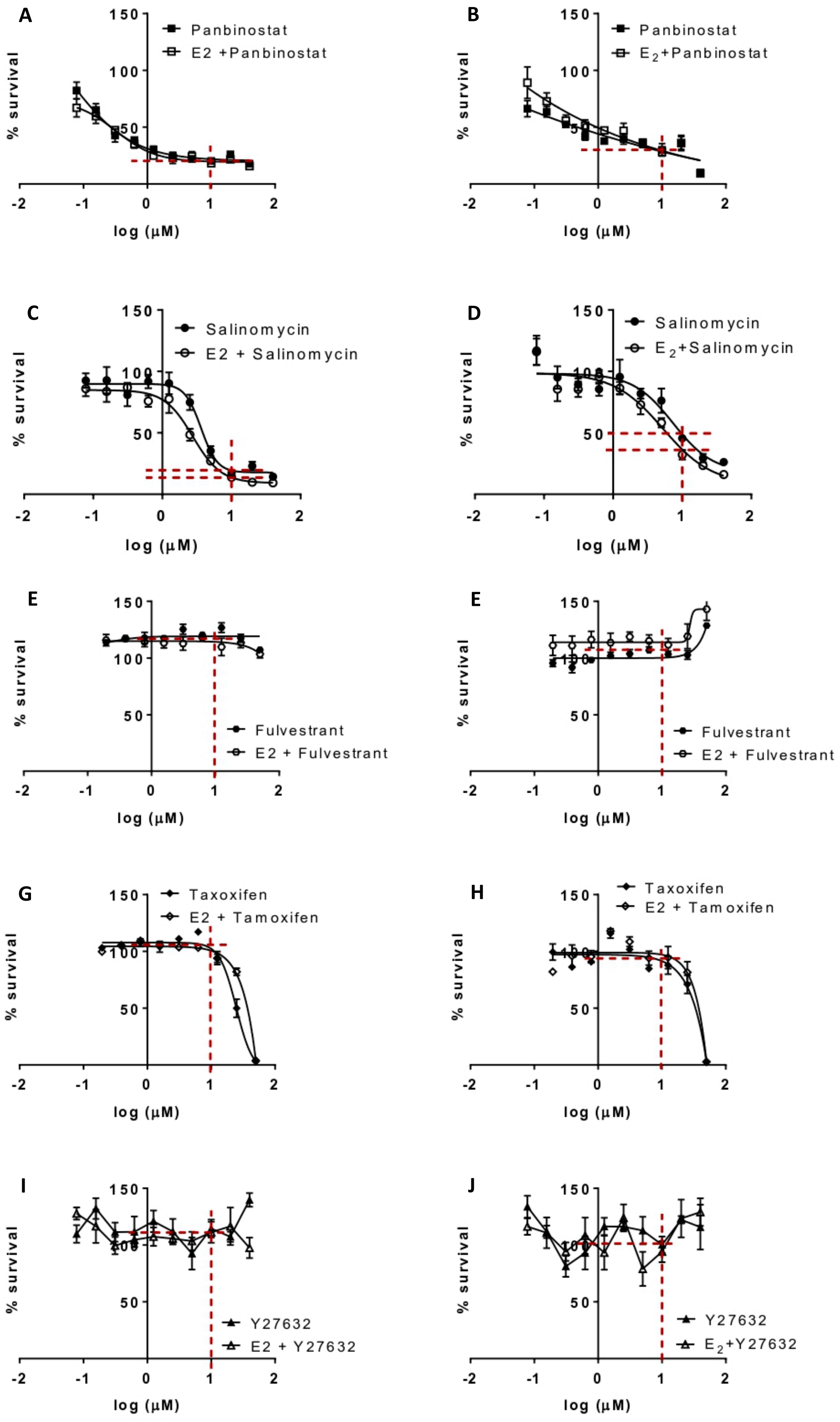

Supplementary Figure S2: Dose response curves for QC compounds. Panobinostat (A-B) salinomycin (C-D), fulvestrant (E-F), tamoxifen (G-H), Y26732 (I-J) inhibition or stimulus of 1455 (A, C, E, G, I) and 1458 (B, D, F, H, J) cell growth in a

dose dependent manner in both vehicle and estrogen conditions. The 10 μM (log 1μM) HTS compound concentration labelled with red dotted line to demonstrate the percentage of cell survival.

Supplemental Table S1: Z′ factors and %CV for confirmation HTS.

	**1455**		**1458**	
	**VEHICLE**	**ESTRADIOL**	**VEHICLE**	**ESTRADIOL**
	**Z′**	**Z′**	**Z′**	**Z′**
**DMSO**				
PANOBINOSTAT	0.51	0.47	0.64	0.64
SALINOMYCIN	0.50	0.46	0.54	0.58
TAMOXIFEN	0.29	0.3	0.73	0.69
**FULVESTRANT**				
PANOBINOSTAT	0.59	0.55	0.65	0.69
SALINOMYCIN	0.58	0.54	0.55	0.64
TAMOXIFEN	0.39	0.39	0.73	0.73
**Y26732**				
PANOBINOSTAT	0.55	0.51	0.66	0.67
SALINOMYCIN	0.55	0.50	0.58	0.63
TAMOXIFEN	0.38	0.38	0.74	0.72
	**%CV**	**%CV**	**%CV**	**%CV**
**Negative Controls**				
DMSO	9.05	10.23	7.43	7.23
FULVESTRANT	7.70	8.62	7.44	5.90
Y26732	11.01	11.80	7.97	5.54
**Positive Controls**				
PANOBINOSTAT	17.35	16.22	18.53	22.31
SALINOMYCIN	13.29	14.72	8.59	10.66
TAMOXIFEN	19.69	24.64	*27.74*	19.29

Supplemental Table S2: Z′ factors and %CV for confirmation HTS.

	1455		1458	
	**VEHICLE**	**ESTRADIOL**	**VEHICLE**	**ESTRADIOL**
	**Z′**	**Z′**	**Z′**	**Z′**
**DMSO**				
SALINOMYCIN	0.51	0.63	0.76	0.60
**FULVESTRANT**				
SALINOMYCIN	0.43	0.53	0.70	0.64
**SNO1004380**				
SALINOMYCIN	0.61	0.65	0.82	0.76
**SNO1006318**				
SALINOMYCIN	0.74	0.67	0.65	0.62
	**%CV**	**%CV**	**%CV**	**%CV**
**Negative Controls**				
**DMSO**	10.07	8.19	5.44	9.04
**FULVESTRANT**	12.24	11.00	6.72	7.45
**SNO1004380**	7.14	7.69	2.85	4.73
**SNO1006318**	3.13	6.86	7.89	9.09
**Positive Control**				
**SALINOMYCIN**	15.64	16.97	16.67	6.57

Supplemental Table S3: Target genes identified from qHTS used for protein-protein-interaction

1455 only	1458 only	Both Cell lines
ABCB1	ABCC4	ADRA1
ALB	ABCC8	ADRA1B
CYP2D6	ABCB11	ADRA1D
CYP3A4	ACE	AHRH1
CYP3A5	ACHE	ATP4A
HRH1	ADRB1	AR
KCNH2	ADRB2	CACNA2D1
NOS3	ALB	CCA
PDG	ANPEP	CHRM1
	APEX1	CHRM2
	AR	CHRM3
	ATF6	CHRM4
	CA1	CHRM5
	CA2	COX2
	CA9	CYP1A2
	CA12	CYP2C9
	CA14	CYP2C19
	CASP1	CYP2D6
	CASP2	CYP3A4
	CASP3	DDAH1
	CASP6	DRD1
	CASP7	DRD2
	CASP8	DRD4
	CCNE1	ESR1
	CCNE2	GABA
	CDK2	GABBR2
	CHRM1	HRH1
	CHRM2	HTR1A

	CHRM4	HTR2A
	CHRM5	HTR2C
	CSNK2A1	IDH1
	CYP2C8	ILIB
	CYP3A4	ISP
	DRD3	KCNMA1
	EGFR	NTRK2
	EHMT1	P53
	EHMT2	SLC1A1
	ESR1	SLC7A5
	ESRRA	TNFA
	FBP1	TRHR
	FOLH1	
	FUCA1	
	GAA	
	GBA2	
	GLB1	
	GPR151	
	HDAC6	
	HIF1A	
	HK1	
	HK2	
	HRH4	
	HTR2A	
	IGF1R	
	KCNH2	
	KCNJ1	
	KCNMA1	
	MAN2A1	
	MAN2B1	

	MAPK1	
	MGAM	
	MME	
	MTAP	
	MTHFR	
	NOS3	
	NR1H4	
	NR1I2	
	NR3C1	
	OGA	
	OPRD1	
	OPRK1	
	PDE5A	
	PDE6G	
	PDE6H	
	PDE10A	
	PDE11A	
	PIK3CG	
	PNP	
	PPARA	
	PPARD	
	PPARG	
	PTGES	
	PYGL	
	RAR	
	RARA	
	RHOB	
	SELP	
	SI	
	SLC5A2	

	SLCO1B1	
	SNAP25	
	TSHR	
	TTR	
	UGCG	
	YARS	
